# ^1^H NMR Serum Metabolomic Change of Trimethylamine N-oxide (TMAO) Is Associated with Alcoholic Liver Disease Progression

**DOI:** 10.3390/metabo14010039

**Published:** 2024-01-08

**Authors:** Junsang Oh, Jayoung Kim, Sanghak Lee, Gyubin Park, Kei-Anne Garcia Baritugo, Ki Jun Han, Sangheun Lee, Gi-Ho Sung

**Affiliations:** 1 Biomedical Institute of Mycological Resource, International St. Mary’s Hospital, College of Medicine, Catholic Kwandong University, Incheon 22711, Republic of Korea; lordjs05@gmail.com (J.O.); lmkjy7@gmail.com (J.K.); 1204keianne@gmail.com (K.-A.G.B.); 2 Department of Convergence Science, College of Medicine, Catholic Kwandong University, Gangneung-si 25601, Gang-won-do, Republic of Korea; 3 Department of Laboratory Medicine, International St. Mary’s Hospital and College of Medicine, Catholic Kwandong University, Incheon 22711, Republic of Korea; 4 Department of Biomedical Science, Graduate School, Catholic Kwandong University, Gangneung-si 25601, Gang-won-do, Republic of Korea; leesh762@gmail.com (S.L.); minhyean1004@gmail.com (G.P.); 5 Department of Internal Medicine, International St. Mary’s Hospital, College of Medicine, Catholic Kwandong University, Incheon 22711, Republic of Korea; 545818@ish.ac.kr

**Keywords:** alcohol-related liver disease, trimethylamine N-oxide, serum metabolites, multivariate analysis, ^1^H-NMR

## Abstract

Without early detection and treatment, chronic and excessive alcohol consumption can lead to the development of alcoholic liver disease (ALD). With this in mind, we exploit the recent concept of the liver–gut axis and analyze the serum profile of ALD patients for identification of microbiome-derived metabolites that can be used as diagnostic biomarkers for onset of ALD. ^1^H-NMR was used to analyze serum metabolites of 38 ALD patients that were grouped according to their Child–Turcotte–Pugh scores (CTP): class A (CTP-A; 19), class B(CTP-B; 10), and class C (CTP-C; 9). A partial least squares-discriminant analysis (PLS-DA) and a variable importance of projection (VIP) score were used to identify significant metabolites. A receiver operating characteristic (ROC) curve and correlation heatmap were used to evaluate the predictability of identified metabolites as ALD biomarkers. Among 42 identified metabolites, 6 were significantly correlated to exacerbation of ALD. As ALD progressed in CTP-C, the levels of trimethylamine N-oxide (TMAO), malate, tyrosine, and 2-hydroxyisovalerate increased, while isobutyrate and isocitrate decreased. Out of six metabolites, elevated levels of TMAO and its precursors (carnitine, betaine, choline) were associated with severity of ALD. This indicates that TMAO can be used as an effective biomarker for the diagnosis of ALD progression.

## 1. Introduction

Liver diseases can be caused by various risk factors such as alcohol consumption, metabolic disorders, viral infection, abnormal immune functions, and genetic predisposition. Because of its global prevalence and high morbidity and mortality, liver disease is considered as one of global health problems [1]. Chronic and excessive alcohol consumption leads to alcoholic liver disease (ALD). ALD is characterized by fatty liver, inflammation, steatosis, fibrosis, cirrhosis, alcoholic hepatitis, and hepatocellular cancer (HCC) [2]. Because the prognosis of ALD is an important factor for effective treatment, non-invasive scoring classification systems have been developed, such as the Model for End-Stage Liver Disease (MELD) score, Child–Turcotte–Pugh (CTP) score, and Sequential Organ Failure Assessment (SOFA) score [3].

Since the gut microbiome and its metabolites are strongly associated with human health and several diseases, extensive research has been conducted to understand and exploit this concept. Among these studies, one of the representative metabolites derived from the gut microbiome is trimethylamine (TMA). TMA is produced in the gut from precursors such as betaine, carnitine, and choline [4,5]. TMA is oxidized into Trimethylamine N-oxide (TMAO) by flavin-dependent monooxygenases (FMOs) in the liver and then released and circulated in the blood [6]. The increase in TMAO levels in the blood is known to be associated with cardiovascular diseases and neurodegenerative diseases, as well as non-alcoholic fatty liver disease (NAFLD) [7,8,9]. In addition, other important microbial metabolites such as short-chain fatty acids (SCFAs) and branched-chain (BCAAs) and aromatic (AAAs) amino acids. BCAAs have also been reported to be involved in the development of diabetes and NAFLD [10]. Although a couple of studies have demonstrated that the intestinal microflora plays an important role in the onset and progression of ALD [11], the exact relationship between gut microbiome derived-metabolites and ALD is still not fully understood. Thus, this paper focuses on the investigation of changes in serum metabolite profile according to the progression of ALD in afflicted patients. This investigation uses ^1^H-NMR metabolomic approach to identify significant metabolites which can be used as biomarkers for diagnosis of ALD liver disease and help discriminate between stages of ALD progression.

## 2. Materials and Methods

### 2.1. Patient Information and Data Collection

The present study was conducted on patients that received treatment for ALD between September 2018 and May 2020 in International St. Mary’s Hospital of Catholic Kwandong University in the Republic of Korea. All patients included in the study were over 19 years old. ALD was defined as intake of chronic excessive alcohol (>50 g of alcohol per day) for more than 6 months and having abnormal blood alanine aminotransferase (ALT), aspartate transaminase (AST), and total bilirubin levels without other chronic liver diseases including chronic hepatitis B, chronic hepatitis C, primary biliary cirrhosis, and Wilson’s disease [12]. Based on CTP (Child–Turcotte–Pugh) score, the baseline characteristics were sub-grouped into CTP class A (CTP-A), CTP class B (CTP-B), and CTP class C (CTP-C). CTP was used as a classification system for assessment of prognosis of patients with liver cirrhosis (LC) [13,14]. It incorporated five variables designed for designating the liver disease progression. The variables include serum albumin, bilirubin, ascites, encephalopathy, and prothrombin time. The score ranges for CTP were from 5 to 15. Patients with a score of 5 or 6 are classified as CTP-A (well-compensated cirrhosis), those with a score of 7 to 9 have CTP-B cirrhosis (significant functional compromise), and those with a score of 10 to 15 have CTP-C cirrhosis (decompensated cirrhosis). The baseline characteristics were expressed as mean ± standard deviation for continuous variables or as percentages for categorical variables. The significance of differences in continuous variables among three groups was analyzed using ANOVA test. The significance of differences in categorical variables was determined using a chi-square test (Table 1).

### 2.2. ^1^H Nuclear Magnetic Resonance (^1^H NMR)

Serum samples of a total of 38 patients were collected and preserved at −80 °C until required. For ^1^H NMR experiment, the frozen serum samples were thawed using a water bath and thoroughly mixed using a vortex mixer. Then, 500 µL of each sample was transferred to a 10 kDa Amicon^®^ Ultra-0.5 Device (Milli-pore, Burlington, MA, USA) and centrifuged at 4 °C and 13,000 rpm for 15 min to eliminate proteins from the serum. Once protein filtering was completed, 300 µL of the resulting serum was combined with 300 µL of PBS buffer (pH 7.4) in a 1.5 mL tube, while keeping the sample on ice. The mixture was mixed well, and then, 600 µL of the sample was transferred into a 5 mm NMR tube for ^1^H NMR measurement using a Bruker 600 MHz spectrometer. The internal standard was used with 0.05 mM of 3-(trimethylsilyl) propionic-2,2,3,3-d4 acid sodium salt (TSP) for calibration of the chemical shift and quantification of metabolites.

### 2.3. ^1^H NMR Data Processing and Multivariate Statistical Analysis

The raw spectral data obtained from the ^1^H NMR experiment was phased, and the baselines were manually corrected using Chenomx NMR Suite software (Version 8.6, Edmonton, AB, Canada). With reference to the internal standard at 0.00 ppm, the ^1^H NMR spectrum was divided into 235 integrated regions by binning the chemical shift to a width of 0.04 ppm, covering a range of 0.0–10.0 ppm. We disregarded the regions of δ 4.60–5.20 ppm, which represented the residual signals of water. The binned data obtained from Chenomx was then imported into the software SIMCA-P+ (version 15.0, Umetrics, Umeå, Sweden). To minimize variations in concentration between samples, normalization by median was applied with auto-scaling (mean-centering and division by the standard deviation of each variable). Once the data were preprocessed, the Principal Component Analysis (PCA) was initially performed as an unsupervised method to identify multivariate outliers and partial least squares discriminant analysis (PLS-DA) was applied to cluster metabolites according to CTP classification to understand the influence of detected metabolites on the liver function. The model’s internal cross-validation was assessed through total variance (R2) and predictive ability value (Q2), and the permutation test was conducted as an external validation. The statistical significance of the predicted power of the PLS-DA model is given by comparing the R2Y and Q2Y values of the original model with randomly permutated models of Y data. Variable importance in the projection (VIP) represents the sum of squares of the PLS-DA weight, considering both Y, which correlates with all responses, and X, which is its projection. The VIP scores derived from the PLS-DA analysis were used to identify the metabolites that have a significant impact on the clustering. Metabolites that achieved values exceeding 0.7 were regarded as highly influential metabolites. Following the identification of these influential metabolites, the concentration of each metabolite was determined using relative intensity (RI), which is based on the peak area relative to the internal standard of TSP. MetaboAnalyst (Version 4.0, http://www.metaboanalyst.ca/, accessed on 15 October 2020) was employed to carry out one-way analysis of variance (ANOVA), which was then followed by post hoc testing using Fisher’s least significant difference (LSD) test.

### 2.4. Receiver Operating Characteristic (ROC) Curve and Heatmap Analyses

Receiver operating characteristic (ROC) curve analysis was conducted to evaluate the predictive values of metabolites for ALD diagnostic biomarkers using MetaboAnalyst (Version 4.0). Reciprocal ROC analyses with three CTP classes were conducted to further evaluate the consistency of the predictive values of selected metabolites. Pearson correlation heatmap was also constructed to evaluate the clustering pattern of selected metabolites based on the interquartile range (IQR) statistical analysis of using MetaboAnalyst (Version 4.0).

## 3. Results

### 3.1. Clinical Characteristics of the Study Population

A total of 38 patients were included in the present study, including 19 patients in CTP-A, 10 patients in CTP-B, and 9 patients in CTP-C. The baseline characteristics of CTP classification, obtained in the present study, are described in Table 1. The levels of albumin, bilirubin, and international normalized ratio (INR), which are considered as important criteria in CTP classification, were statistically different among patients in CTP classes.

**Table 1:** Baseline characteristics of patients classified according to CTP classification. Continuous variables are expressed as means and standard deviations and categorical variables are expressed as numbers and percentages.

	CTP-A (*n* = 19)	CTP-B (*n* = 10)	CTP-C (*n* = 9)	*p*-Value
Age (years)	55.5 (45–73)	51 (34–66)	51 (41–68)	0.36500
Gender (Male)	17 (89.5)	9 (80.0)	6 (66.7)	0.34363
Hypertension	7 (36.8)	3 (30.0)	2 (22.2)	0.73352
Diabetes Mellitus	4 (21.1)	1 (9.1)	2 (5.3)	0.72398
Hyperlipidemia	3 (15.8)	1 (10.0)	1 (11.1)	0.88895
BMI (kg/m^2^)	23.6 (16.8–29.7)	21.9 (16.5–32)	23.1 (18.7–30.1)	0.64900
Stiffness (kPa)	11.0 (4.2–75)	48.4 (5.6–75)	44.6 (17.6–67.8)	0.06324
Total bilirubin (mg/dL)	1.0 (0.4–4.0)	2.95 (0.8–20.6)	5.4 (1.2–38.1)	0.00017
Albumin (mg/dL)	3.6 (3.1–4.6)	2.95 (2.0–4.7)	2.5 (1.8–3.4)	0.00004
AST (IU/dL)	55 (14–3387)	76 (18–789)	89 (31–331)	0.57530
ALT (IU/dL)	46 (8–2835)	46.5 (10–2080)	26 (11–66)	0.62040
GGT (mg/dL)	218 (22–2760)	138 (22–884)	146 (16–477)	0.57350
Platelet (10^3^/μL)	182 (54–372)	110 (55–247)	62 (20–139)	0.00203
INR	1.05 (0.89–1.55)	1.37 (0.98–1.91)	1.55 (1.31–2.26)	0.00005
Creatinine	0.92 (0.16–4.67)	0.72 (0.32–6.22)	0.77 (0.39–4.41)	0.55880

BMI: body mass index; AST: aspartate aminotransferase; ALT: alanine aminotransferase; GGT: gamma-glutamyl transferase; INR: international normalized ratio.

### 3.2. Identification of Serum Metabolites in ALD Patients by ^1^H-NMR

In the present study, metabolites were identified by peak fitting with the Chenomx database after the chemical shift calibration with TSP. A total of 42 metabolites were identified, with the majority belonging to primary metabolites, including one sugar, two sugar alcohols, two amines, 14 amino acids, and 23 organic acids, as described in Table 2. In Fisher’s Least Significant Difference (LSD) test based on one-way analysis of variance (ANOVA), IRs of six metabolites (TMAO, malate, isobutyrate, tyrosine, 2-hydroxyisovalerate, and valine) of CTP-B and CTP-C were statistically different from those of CTP-A.

### 3.3. Multivariate Analyses of Serum Metabolite Data from ALD Patients According to CTP Classes

**Figure 1:**
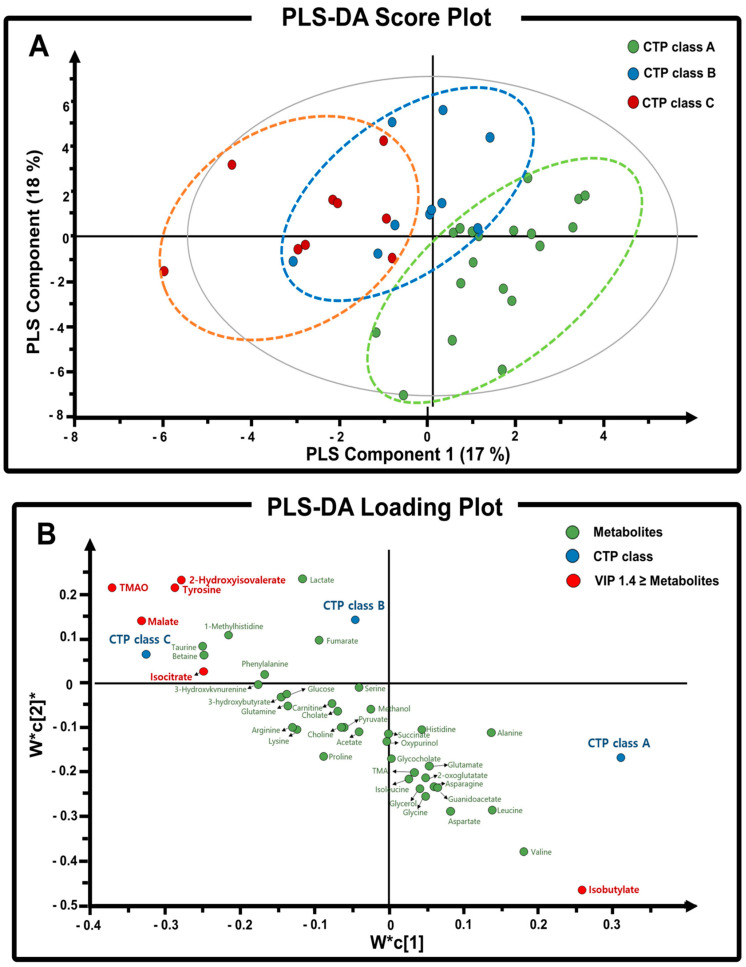
(**A**) PLS-DA score plot of serum samples of CTP-A, CTP-B, and CTP-C based on the CTP classification of ALD disease progression. (**B**) PLS-DA loading plot of serum metabolites in ALD. The two components (W*c [2]/W*c [1]) explain the separation among classes. Metabolites which are important in separation among CTP classes are highlighted in red with VIP score cut-off value of 1.4. The loading plot is complementary to the score plot and summarizes how the X-variables relate to each other as well as to group belonging (Y-variable symbolized by a class dot). X-variables located near a class dot are positively associated with that class.

The multivariate statistical methods such as an unsupervised PCA and a supervised PLS-DA analysis have been widely used in metabolomic studies for the pattern recognition and the prediction of diagnostic biomarkers. Based on the ^1^H-NMR data, we performed PCA and PLS-DA analyses to identify metabolites associated with the corresponding CTP class. The results of PCA score plot (Supplementary Figure S1) showed the separation of partial overlaps based on principal component 1 (PC1), which explained 27.3% of the variance. As compared with PCA score plot, PLS-DA score plot showed a clear separation among CTP classes (Figure 1A). In addition, PLS-DA loading plot analyses showed six important metabolites (TMAO, 2-hydroxyisovalerate, tyrosine, malate, isocitrate, and isobutyrate) contributing to the CTP class separation (Figure 1B). We used the VIP score value in PLS-DA loading plot to identify these crucially important metabolites of the CTP class separation (Supplementary Table S1). For the VIP 1.4 score value of loading plot, the permutation test was performed 999 times, and the optimized parameters were selected by using the R2Y (0.621) and Q2Y (0.539) values (Supplementary Table S2). Based on the results of the separation of the CTP class in ALD liver disease progression with a VIP score cut-off value of 1.4, six metabolites (TMAO, malate, isobutyrate, tyrosine, 2-hydroxyisovalerate, isocitrate) were associated with the separation of metabolites among CTP classes.

**Figure 2:**
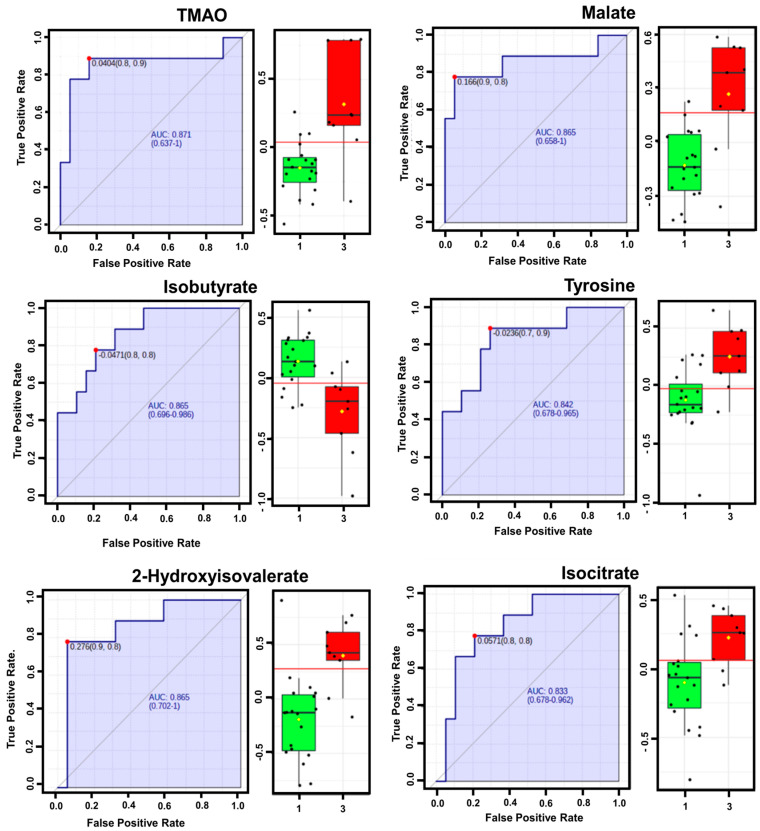
ROC curve graph and the log-transformed RI of six metabolites which were useful in predicting the liver disease progression in AUC analyses between CTP-A and CTP-C. For each metabolite, the left side is ROC curve graph, and the right side is the boxplots of log-transformed RI of CTP-A (boxplot in green) and CTP-C (boxplot in red).

To evaluate the predictive values of six influential metabolites (TMAO, malate, isobutyrate, tyrosine, 2-hydroxyisovalerate, isocitrate) in the PLS-DA loading plot analyses, the reciprocal ROC curve analyses were performed with three CTP classes in ALD disease progression. In the ROC comparison between CTP-A and CTP-C, the six metabolites identified in loading plot analyses with a VIP score cut-off value of 1.4 were also predicted as potential biomarkers with the area under the ROC curve (AUC) values ranging from 0.833 to 0.871 (Figure 2 and Supplementary Table S3). As a result, the six metabolites that are identified from both the loading plot and ROC analyses were TMAO, malate, iso-butyrate, tyrosine, 2-hydroxyisovalerate, and isocitrate, which can be considered as potential biomarkers in ALD liver disease progression. Although these metabolites were not predictive in the ROC comparison between CTP-B and CTP-C, they were also ranked as relatively important in the ROC comparison between CTP-A and CTP-B (Tables S4 and S5). As a result, considering the results of both the loading plot analyses and ROC curve analyses, these six metabolites can be considered as potential biomarker candidates of metabolic changes in the liver disease progression of ALD.

**Figure 3:**
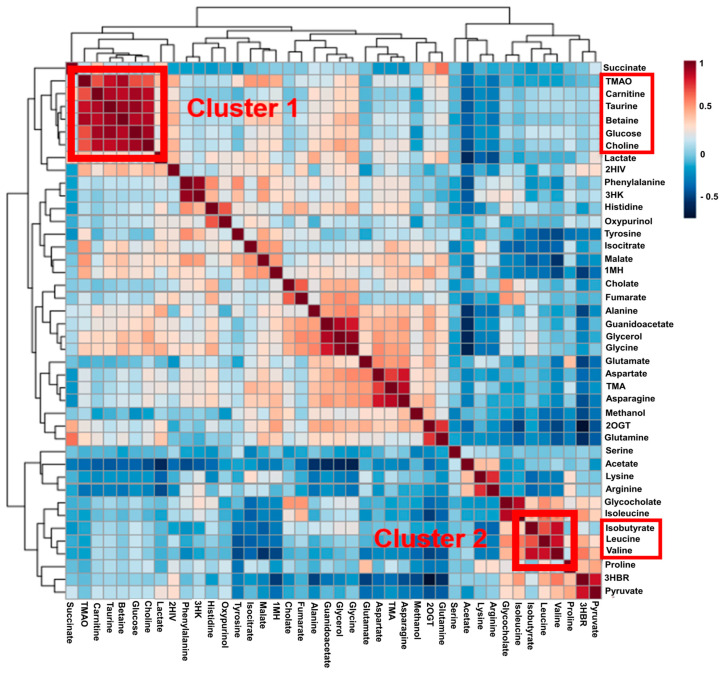
Pearson correlation heatmap for the clustering of significant metabolites in serum samples. Pearson distance measurements were used based on the interquartile range. Out of six influential metabolites (TMAO, malate, isobutyrate, tyrosine, 2-hydroxyisovalerate, isocitrate), TMAO was included in Cluster 1, and isobutyrate was included in Cluster 2.

To identify metabolites can be correlated with these six metabolites (TMAO, malate, isobutyrate, tyrosine, 2-hydroxyisovalerate, and isocitrate) in the metabolic process of liver disease progression, we used a correlation matrix heatmap construct by clustering a total of 42 metabolites identified in the present study using supervised hierarchical clustering (Figure 2). The two clusters related with TMAO and isobutyrate were found to be statistically significant (*p* < 0.005 and *p* < 0.01, respectively). The metabolites correlated with TMAO in Cluster 1 were carnitine, taurine, betaine, glucose, and choline. Leucine and valine were correlated with isobutyrate in Cluster 2 in Figure 3. Like TMAO, the concentration of TMAO precursors, including carnitine, betaine, and choline (all of them are metabolites related with intestinal environment), also increased.

## 4. Discussion

Although alcohol abuse is generally considered as a psychiatric disorder, alcoholism is closely related to dysbiosis in the gut microbiome [11]. For example, excessive alcohol consumption reduces the population of beneficial commensal bacteria such as *Bifidobacterium* and *Lactobacillus*. Alcohol also decreases the intestinal levels of long-chain fatty acids, which are used by commensal gut microbiota as their main energy source [15,16]. On the other hand, several studies have suggested that the intestinal microbiome and its metabolites can also play an important role as biomarkers for the diagnosis of liver disease progression [17]. Without early detection, chronic alcohol consumption significantly contributes to the onset and progression of severe liver diseases such as ALD and HCC. Recently, several studies have focused on the identification of key metabolites which can be used to diagnose the progression of liver diseases such as NAFLD, nonalcoholic steatohepatitis (NASH), LC, and HCC for diagnosis and treatment. However, analysis of key metabolites present at early stages and progression of ALD has not been conducted.

Thus, in this study, we focused on the analysis of serum metabolic profiles related to ALD liver disease progression in 38 patients. The patients were grouped into three groups according to their respective CTP classification. Based on the PLS-DA modeling, we calculated VIP scores for comparison of metabolite levels and identification of metabolites that highly contribute to CTP class discrimination. Significant metabolites observed include TMAO, malate, isobutyrate, tyrosine, 2-hydroxyisovalerate, and isocitrate. These six metabolites were strongly associated with the progression of ALD (Figure 1). These metabolites were also found to be important according to ROC analyses, which evaluated whether these six metabolites can be used as diagnostic biomarkers for the progression of ALD (Figure 2).

Among the six significant metabolites, TMAO was deemed to be of most interest because it is representative of gut microbiome-derived metabolites. TMAO is produced by the oxidation of TMA, which is derived from the main TMAO precursors, choline, betaine, and carnitine. In the present study, the progression of ALD was strongly associated with elevated levels of TMAO and its precursors. Several studies have indicated that serum levels of TMAO are correlated to changes in the gut microflora, diet, and FMO activity. On the other hand, elevated levels of TMAO have also been reported to indicate the onset of diseases related to neurodegenerative, cardiovascular, and liver systems. Thus, TMAO is considered as an important biomarker and a new therapeutic target for many associated diseases [6,18,19,20]. For example, Chen et al. [19] found that increased plasma TMAO levels were correlated with the severity of NAFLD in humans. On the other hand, Dumas et al. [20] also showed that increased urinary excretion of methylamines, such as TMA, TMAO, and dimethylamine, was linked to the development of NAFLD in mice. In the case of ALD in the present study, we found that the level of serum TMAO significantly increased as classification shifted from CTP-A to CTP-C. This suggests that TMAO levels are highly correlated with the progression of ALD (Figure 1B and Table 1).

On the other hand, the concentration of isobutyrate dramatically decreased as classification shifted from CTP-A to CTP-C in the present study (Figure 2 and Figure 3). Isobutyrate is another important microbial metabolite derived from SCFAs (acetate, propionate, and butyrate). SCFAs regulate a variety of biological functions including glucose and lipid metabolism and immune function in the liver. These biological activities are mediated by the activation of G-protein coupled receptors and inhibition of histone deacetylase [21]. For example, a clinical study demonstrated that the supplementation of propionate to the colon reduced weight gain and intrahepatocellular lipid content [22]. In another study, Zhou et al. [23] found that butyrate produced by the probiotic *Clostridium butyricum* B1 significantly attenuated high-fat diet-induced obesity and steatohepatitis and restored enterohepatic immune disorder in mice. SCFAs are considered as beneficial prebiotics that improve health. However, in contrast to previous reports, Singh et al. [24] demonstrated that a decrease in levels of intestinal SCFAs was associated with the prevention of HCC. Thus, further studies are needed to confirm the relationship between levels of SCFAs and the progression of ALD liver disease.

A heatmap analysis of the metabolite–metabolite correlation matrix identified metabolites that clustered according to the CTP classification or progression of ALD. Interestingly, Cluster 1 includes TMAO precursors (betaine, carnitine, choline), which indicates a positive correlation with TMAO. There are conflicting opinions regarding the effect of carnitine, betaine, and choline on liver diseases. Most studies have suggested that a higher intake or higher plasma levels of carnitine, betaine, and choline may improve fatty liver and liver damage, including NAFLD [25,26]. However, intake of carnitine, betaine, and choline can elevate TMAO levels, which increases the risks of developing associated diseases [27]. Our results suggest that the elevated levels of TMAO and its precursors in the serum are associated with the progression of ALD. This indicates that increased levels of TMAO precursors may contribute to advanced progression of ALD (Figure 2 and Figure 3).

Meanwhile, it was observed that isobutyrate decreased together with BCAAs valine and leucine in Cluster 2 (Figure 3). Lower levels of isobutyrate may be attributed to decreased abundance of SCFA-producing bacteria in the gut because of alcohol-induced gut microbiome dysbiosis. On the other hand, BCAAs are considered as essential nutrients because of their involvement in energy and muscle metabolism and in stress management. Several studies assigned valine as a biomarker for Alzheimer’s disease [16], colorectal cancer [17], and Crohn’s disease [23,28]. In this study, the lower levels of BCAAs may be due to ammonia detoxification and increased activity of branched-chain keto acid dehydrogenase. This phenomena has been associated with diseases such as cirrhosis, urea cycle disorder, and chronic renal failure [29]. Lower levels of BCAAs are characteristic of advanced liver disease, and long-term supplementation of BCAAs can be applied for an improvement of liver function and reduction of cirrhosis in afflicted patients.

## 5. Conclusions

In conclusion, we used the ^1^H-NMR metabolomics approach to profile serum metabolites of ALD patients to identify metabolites which can be used as biomarkers for the progression of ALD. Among 42 metabolites identified, six metabolites (TMAO, malate, isobutyrate, tyrosine, 2-hydroxyisovalerate, and isocitrate) were statistically different between CTP classes and can be used to correlate the progression of ALD from CTP-A to CTP-C, according to PLS-DA, VIP score, and ROC curve analyses. Therefore, these metabolites can be used as biomarkers for the diagnosis of severity of ALD. TMAO is recommended as the best candidate to use as a biomarker because of the significant correlation observed between elevated TMAO levels and its precursors (carnitine, betaine, choline) during progression of ALD, and further studies are necessary to validate our results with larger sampling based on CTP classification.

## Figures and Tables

**Table 2:** Identification and quantitative comparison of serum metabolites between classes in ALD disease progression. The chemical shift and the relative intensity (RI) representing the concentration of each metabolite were shown as the mean and standard deviation.

HMDB Card	Metabolites	Chemical Shifts (Multiplicities) (ppm)	CTP-A Mean ± SD	CTA-B Mean ± SD	CTA-C Mean ± SD
HMDB0000042	Acetate	1.9 (s)	0.056 ± 0.019	0.051 ± 0.028	0.057 ± 0.026
HMDB0001310	Alanine	1.46 (q)	0.131 ± 0.033	0.137 ± 0.031	0.109 ± 0.025
HMDB0000517	Arginine	1.86 (m)	0.085 ± 0.027	0.070 ± 0.027	0.111 ± 0.057
HMDB0000168	Asparagine	2.86–2.94 (t)	0.043 ± 0.013	0.039 ± 0.013	0.041 ± 0.012
HMDB0000191	Aspartate	2.78–2.82 (dd)	0.041 ± 0.011	0.035 ± 0.010	0.038 ± 0.012
HMDB0000043	Betaine	3.26 (s), 3.90 (s)	0.527 ± 0.117	0.607 ± 0.174	0.714 ± 0.259
HMDB0000062	Carnitine	2.42–2.46 (m), 3.22 (s)	0.524 ± 0.092	0.579 ± 0.161	0.603 ± 0.227
HMDB0000619	Cholate	0.70 (s)	0.017 ± 0.007	0.015 ± 0.004	0.019 ± 0.007
HMDB0000097	Choline	3.18 (s), 3.50 (dd)	0.317 ± 0.089	0.355 ± 0.121	0.380 ± 0.169
HMDB0000134	Fumarate	6.50 (s)	0.012 ± 0.005	0.013 ± 0.003	0.013 ± 0.005
HMDB0000122	Glucose	3.38 (dd), 3.50 (dd), 3.70–3.72 (m), 3.82–3.86 (m)	2.024 ± 0.627	2.245 ± 0.781	2.501 ± 1.179
HMDB0003339	Glutamate	2.02–2.06 (m), 2.34 (m)	0.100 ± 0.033	0.087 ± 0.027	0.095 ± 0.047
HMDB0000641	Glutamine	2.14–2.18 (m), 2.42 (m)	0.188 ± 0.037	0.191 ± 0.048	0.199 ± 0.058
HMDB0000131	Glycerol	3.54 (dd), 3.66 (dd), 3.78 (m)	15.206 ± 2.789	14.336 ± 2.420	14.926 ± 2.274
HMDB0000123	Glycine	3.54 (s)	5.149 ± 0.690	4.906 ± 0.664	5.067 ± 0.487
HMDB0000138	Glycocholate	0.74 (s), 0.90(s)	0.072 ± 0.019	0.063 ± 0.016	0.075 ± 0.016
HMDB0000128	Guanidoacetate	3.78 (s)	4.355 ± 1.105	3.960 ± 0.748	4.179 ± 0.663
HMDB0000177	Histidine	7.22 (s), 8.3 (s)	0.031 ± 0.009	0.030 ± 0.005	0.029 ± 0.007
HMDB0000011	3-Hydroxybutyrate	1.18–1.22 (d), 2.3 (dd)	0.132 ± 0.100	0.079 ± 0.035	0.183 ± 0.198
HMDB0000407	2-Hydroxyisovalerate	0.82 (d)	0.020 ± 0.013	0.030 ± 0.017 ^a^	0.036 ± 0.012 ^a^
HMDB0011631	3-Hydroxykynurenine	7.42–7.48 (dd)	0.030 ± 0.007	0.028 ± 0.008	0.037 ± 0.012
HMDB0001873	Isobutyrate	1.02–1.04 (d)	0.085 ± 0.021	0.061 ± 0.015 ^a^	0.066 ± 0.018 ^a^
HMDB0000193	Isocitrate	2.50–2.54 (dd), 2.96–3.06 (m)	0.084 ± 0.012	0.088 ± 0.028	0.110 ± 0.016
HMDB0000172	Isoleucine	0.90–0.93 (t), 1.26 (m)	0.074 ± 0.022	0.062 ± 0.015	0.074 ± 0.016
HMDB0000190	Lactate	1.30 (d), 4.10 (q)	0.565 ± 0.129	0.710 ± 0.229	0.622 ± 0.204
HMDB0000687	Leucine	0.94 (m), 1.66–170 (m)	0.126 ± 0.042	0.103 ± 0.031	0.108 ± 0.038
HMDB0000182	Lysine	1.7 (m), 1.86–1.94 (m), 3.02 (t)	0.211 ± 0.066	0.191 ± 0.064	0.254 ± 0.067
HMDB0000156	Malate	2.66 (dd)	0.038 ± 0.008	0.041 ± 0.009 ^a^	0.051 ± 0.008 ^a^
HMDB0001875	Methanol	3.34 (s)	0.062 ± 0.018	0.058 ± 0.022	0.064 ± 0.015
HMDB0000001	1-Methylhistidine	3.10 (dd), 7.78 (s)	0.045 ± 0.014	0.053 ± 0.015	0.056 ± 0.007
HMDB0000208	2-Oxoglutatate	2.43–2.46 (t), 2.98–3.0 (t)	0.082 ± 0.019	0.081 ± 0.017	0.078 ± 0.019
HMDB0000786	Oxypurinol	8.26(s)	0.016 ± 0.006	0.014 ± 0.004	0.016 ± 0.003
HMDB0000159	Phenylalanine	7.3 (m), 7.42 (m)	0.054 ± 0.010	0.055 ± 0.013	0.063 ± 0.014
HMDB0000162	Proline	2.20 (m)	0.103 ± 0.030	0.089 ± 0.021	0.118 ± 0.036
HMDB0000243	Pyruvate	2.38 (s)	0.042 ± 0.015	0.034 ± 0.008	0.049 ± 0.031
HMDB0000187	Serine	3.94–3.98 (dd)	0.078 ± 0.037	0.081 ± 0.027	0.083 ± 0.039
HMDB0000254	Succinate	2.39–2.42 (t)	0.077 ± 0.013	0.076 ± 0.020	0.081 ± 0.021
HMDB0000251	Taurine	3.26 (t), 3.42 (t)	0.582 ± 0.133	0.682 ± 0.207	0.822 ± 0.352
HMDB0000906	TMA	2.90 (s)	0.025 ± 0.007	0.022 ± 0.009	0.025 ± 0.007
HMDB0000925	TMAO	3.26 (s)	0.201 ± 0.035	0.246 ± 0.064 ^a^	0.316 ± 0.095 ^a^
HMDB0000158	Tyrosine	6.90 (ddd), 7.18 (ddd)	0.053 ± 0.009	0.061 ± 0.024 ^a^	0.073 ± 0.016 ^a^
HMDB0000883	Valine	0.98 (m), 0.99–1.02 (dd), 2.26 (m)	0.235 ± 0.076	0.163 ± 0.032 ^a^	0.190 ± 0.093 ^a^

HMDB: Human Metabolome Database; ^a^ statistically different from CTP-A in LSD test with *p*-value < 0.05.

## Data Availability

Data presented in this study are available on request from the corresponding author. Data available on request due to ethical restriction.

## Supplementary Materials

The following supporting information can be downloaded at: https://www.mdpi.com/article/10.3390/metabo14010039/s1, Figure S1: PCA score plot of serum from ALD patients according to CTP classification; Table S1: VIP score of serum metabolites using the optimized PLS model; Table S2: Parameters from permutation test of PLS-DA models derived from various VIP cut-off values; Table S3: ROC analysis of serum metabolites to compare CTP class A and class C; Table S4. ROC analysis of serum metabolites to compare CTP class A and class B; Table S5. ROC analysis of serum metabolites to compare CTP class B and class C.
