# Supplementary material for: 1H NMR Serum Metabolomic Change of Trimethylamine N-oxide (TMAO) Is Associated with Alcoholic Liver Disease Progression
Source: Metabolites. 2024 Jan 8;14(1):39. doi: 10.3390/metabo14010039 (PMC10818766; doi:10.3390/metabo14010039)
Supplement: Supplementary file 1 [file metabolites-14-00039-s001.zip › metabolites-2743156-supplementary.pdf]

Supplementary Figure S1. PCA score plot of serum from ALD patients according to CTP classification.

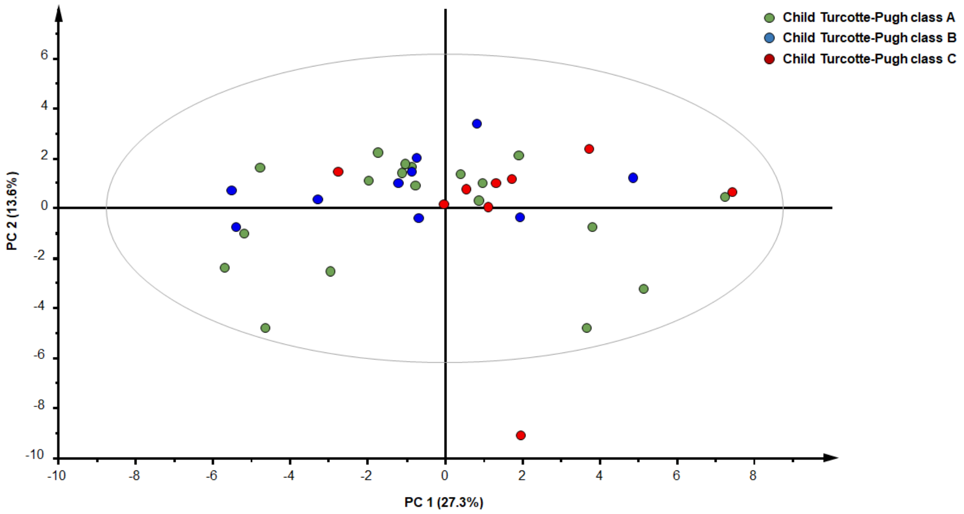

Supplementary Table S1. VIP score of serum metabolites using the optimized PLS model.

Number	Metabolites	VIP Score
1	TMAO	2.01439
2	Malate	1.82034
3	Isobutyrate	1.78457
4	Tyrosine	1.56147
5	2-Hydroxyisovalerate	1.51803
6	Isocitrate	1.42660
7	Betaine	1.38775
8	Taurine	1.38672
9	Valine	1.37450
10	1-Methylhistidine	1.16860
11	Leucine	1.04756
12	3-Hydroxykynurenine	1.02633
13	Aspartate	0.96292
14	Arginine	0.95248
15	Phenylalanine	0.95239
16	Lysine	0.93355
17	Proline	0.91881
18	Glucose	0.89987
19	Choline	0.88390
20	Glycine	0.84607
21	Carnitine	0.83875
22	Lactate	0.83551
23	Glycerol	0.79663
24	Guanidoacetate	0.78919

25	Asparagine	0.77668
26	Alanine	0.76710
27	TMA	0.73531
28	Glutamate	0.70986
29	Isoleucine	0.67752
30	2-Oxoglutatate	0.62770
31	Glycocholate	0.60840
32	Glutamine	0.60468
33	Pyruvate	0.59050
34	3-Hydroxykynurenine	0.53477
35	Cholate	0.53273
36	Succinate	0.52979
37	Fumarate	0.51269
38	Acetate	0.48609
39	Oxypurinol	0.41296
40	Histidine	0.38157
41	Methanol	0.29770
42	Serine	0.24594

Supplementary Table S2. Parameters from permutation test of PLS-DA models derived from various VIP cut-off values. If R2 values exceeded 0.5 and were greater than Q2 values, the model was deemed valid. The model is considered valid if the R2Y intercept falls below 0.3–0.4 and the Q2Y intercept is under 0.05.

VIP cut-off	metabolites	R^2Y	Q^2Y	R^2Y intercept	Q^2Y intercept
0.6	32	0.432	0.275	0.177	-0.232
0.7	28	0.439	0.298	0.159	-0.241
0.8	22	0.417	0.291	0.135	-0.234
0.9	17	0.428	0.315	0.127	-0.225
1.0	12	0.556	0.450	0.103	-0.223
1.1	10	0.586	0.500	0.077	-0.223
1.2	9	0.587	0.501	0.063	-0.222
1.4	6	0.621	0.539	0.054	-0.227

Supplementary Table S3. ROC analysis of serum metabolites to compare CTP class A and class C.

Metabolites	AUC	P value	Fold Change
TMAO	0.871249	0.000296	-0.609790
Isobutyrate	0.865249	0.000326	-0.434990
2-Hydroxyisovalerate	0.865249	0.000613	-0.818210
Malate	0.865249	0.000711	0.378714
Tyrosine	0.842257	0.002507	-0.457450
Isocitrate	0.833461	0.009678	-0.406500
Valine	0.795322	0.012783	0.355839
Taurine	0.748538	0.015085	-0.431060
Leucine	0.730994	0.037655	0.235521
Betaine	0.730994	0.018489	-0.393030

1-Methylhistidine	0.713450	0.059070	-0.351810
Alanine	0.701754	0.056992	0.248049
Lysine	0.684211	0.093231	-0.275460
3-Hydroxybutyrate	0.654971	0.372316	-0.380270
2-Oxoglutatate	0.649123	0.336757	0.070514
Arginine	0.631579	0.149560	-0.300790
3-Hydroxykynurenine	0.608187	0.124220	-0.271770
Glucose	0.602339	0.235291	-0.251680
Glycerol	0.602339	0.337537	0.018865
Proline	0.590643	0.317781	-0.195800
Glycine	0.590643	0.291960	0.016668
Histidine	0.584795	0.653018	0.037353
Glutamate	0.578947	0.298829	0.170587
Phenylalanine	0.573099	0.199704	-0.208540
Acetate	0.573099	0.758335	-0.013340
Guanidoacetate	0.573099	0.422998	0.032831
Cholate	0.567251	0.524494	-0.177590
Isoleucine	0.561404	0.755531	-0.025000
Asparagine	0.561404	0.429534	0.080188
Oxypurinol	0.549708	0.541969	-0.056390
Lactate	0.543860	0.773830	-0.116820
Aspartate	0.543860	0.298340	0.126686
Fumarate	0.543860	0.500284	-0.136440
Choline	0.543860	0.321957	-0.212920
TMA	0.526316	0.674676	0.006361
Pyruvate	0.514620	0.710996	-0.140380
Serine	0.514620	0.645388	-0.111990
Glycocholate	0.508772	0.944799	-0.054690
Glutamine	0.508772	0.955132	-0.060690
Methanol	0.502924	0.854979	-0.094150
Succinate	0.502924	0.695224	-0.030350
Carnitine	0.502924	0.460488	-0.143660

Supplementary Table S4. ROC analysis of serum metabolites to compare CTP class A and class B.

Metabolites	AUC	P value	Fold Change
Valine	0.82105	0.00431	0.52905
Isobutyrate	0.8	0.00245	0.46405
2-Hydroxyisovalerate	0.794737	0.012213	-0.57098
Malate	0.778947	0.006256	-0.1177
1-Methylhistidine	0.778947	0.019309	-0.2316
TMAO	0.768421	0.007886	-0.2942
Lactate	0.710526	0.039644	-0.32992
Tyrosine	0.705263	0.039814	-0.21606
Taurine	0.7	0.034696	-0.22774
Glutamine	0.668421	0.106374	-0.027164
Betaine	0.663158	0.045536	-0.2033
Succinate	0.652632	0.62013	0.029565
3-hydroxybutyrate	0.647368	0.16057	0.7434
Phenylalanine	0.636842	0.158392	-0.021115
Glycocholate	0.631579	0.506483	0.20711
Arginine	0.631579	0.105404	0.28363
Fumarate	0.631579	0.257923	-0.11737
isoleucine	0.626316	0.32448	0.25155
isocitrate	0.626316	0.255685	-0.063728
Alanine	0.605263	0.247032	-0.06489

Glucose	0.605263	0.136674	-0.14971
Choline	0.6	0.106556	-0.16145
Pyruvate	0.594737	0.403983	0.3159
Carnitine	0.589474	0.078912	-0.14248
TMA	0.589474	0.436809	0.19596
Cholate	0.584211	0.946803	0.18697
Serine	0.584211	0.467657	-0.055098
Asparagine	0.578947	0.769454	0.14871
Aspartate	0.578947	0.457313	0.20546
Leucine	0.573684	0.21785	0.29189
Proline	0.568421	0.453804	0.20709
2-oxoglutatate	0.563158	0.382716	0.019011
Glycine	0.563158	0.611898	0.069802
Glutamate	0.557895	0.570206	0.19265
Oxypurinol	0.547368	0.519414	0.12372
Guanidoacetate	0.547368	0.951221	0.13707
Glycerol	0.547368	0.757619	0.085013
Acetate	0.536842	0.414429	0.12662
Histidine	0.531579	0.384253	0.022719
Lysine	0.526316	0.607451	0.14207
3-Hydroxykynurenine	0.515789	0.6285	0.057618
Methanol	0.510526	0.996954	0.083046

Supplementary Table S5. ROC analysis of serum metabolites to compare CTP class B and class C.

**Metabolites**	**AUC**	**P value**	**Fold Change**
Alanine	0.811111	0.023938	0.325520
3-hydroxybutyrate	0.800000	0.043809	-1.214300
2-oxoglutatate	0.777778	0.037440	0.059541
isocitrate	0.722222	0.194042	-0.316340
Arginine	0.722222	0.073215	-0.663710
Malate	0.711111	0.200600	-0.324890
Lysine	0.700000	0.133255	-0.407210
Histidine	0.688889	0.161563	0.042734
Succinate	0.677778	0.552268	-0.090942
Guanidoacetate	0.677778	0.484068	-0.077440
Glycerol	0.677778	0.361132	-0.058170
Glycine	0.677778	0.278621	-0.046765
Lactate	0.666667	0.165868	0.190050
Cholate	0.655556	0.447237	-0.399990
Leucine	0.644444	0.584713	-0.059809
Glutamine	0.644444	0.253266	-0.058968
TMAO	0.633333	0.473030	-0.360990
Glycocholate	0.633333	0.587676	-0.249030
2-Hydroxyisovalerate	0.633333	0.450234	-0.296920
Pyruvate	0.633333	0.322941	-0.544070
Proline	0.622222	0.165043	-0.409370
isoleucine	0.600000	0.551199	-0.248350
Valine	0.600000	0.821485	-0.221940
Choline	0.600000	0.657629	-0.099450
Isobutylate	0.588889	0.534800	-0.093094
Tyrosine	0.588889	0.383433	-0.260870
Carnitine	0.588889	0.490539	-0.058966
Aspartate	0.566667	0.661229	-0.104220
Glutamate	0.566667	0.585465	-0.122210

Fumarate	0.566667	0.540140	-0.077136
3-Hydroxykynurenine	0.566667	0.361962	-0.373130
TMA	0.555556	0.809290	-0.188920
1-Methylhistidine	0.555556	0.601936	-0.076356
Taurine	0.544444	0.847011	-0.270100
Betaine	0.544444	0.882898	-0.234360
Serine	0.544444	0.392849	-0.035399
Methanol	0.533333	0.843785	-0.144880
Glucose	0.533333	0.809048	-0.155590
Acetate	0.522222	0.656530	-0.160960
Asparagine	0.511111	0.665007	-0.067071
Phenylalanine	0.511111	0.889394	-0.193470
Oxypurinol	0.511111	0.999294	-0.151850
